# Comparative assessment of drying kinetics and physicochemical properties of vegetables under different solar drying methods

**DOI:** 10.1016/j.fochx.2026.103726

**Published:** 2026-03-04

**Authors:** Seid Reza Falsafi, Ramin Gooruee, Hamid Reza Gazor

**Affiliations:** a Food Science and Technology Division, Agricultural Engineering Research Department, Safiabad Agricultural and Natural Resources Research and Education Center, (AREEO), Dezful, Iran; b Agricultural Engineering Research Institute. Karaj, Islamic Republic of Iran

**Keywords:** Solar tent drying, Vegetables, Drying kinetics, Quality attributes, Antioxidant retention

## Abstract

This study compared drying kinetics, physicochemical quality, and sensory attributes of onion (*Allium cepa* L.) and mint (*Mentha piperita* L.) processed by solar tent drying (STD), sun drying (SD), and shade drying (ShD). According to the results, SD provided the fastest drying rate, followed by STD and ShD, whereas ShD preserved the highest total phenolic content (onion: 65.67 mg GAE/g; mint: 70.36 mg GAE/g), antioxidant activity (DPPH: onion 31.10%; mint 98.49%), and rehydration capacity (onion 272.2%; mint 60.87%). STD provided a balanced performance, preserving moderate bioactive content, acceptable color (ΔE = 12.15), and lower microbial loads (1.66 × 10^3^ CFU/g) while shortening drying duration compared with ShD. In contrast, SD caused severe discoloration (ΔE = 30.34), greater shrinkage, and higher microbial counts. PCA confirmed distinct clustering of drying methods, with STD positioned between SD and ShD. Overall, STD represents a practical approach that improves efficiency while maintaining quality.

## Highlights

• Solar tent drying balances quality retention with practical drying efficiency.
• Tent drying preserves color and bioactives better than open sun drying.
• Multivariate analysis confirms drying method as the main quality driver.
• Shade drying maximizes quality but lacks operational feasibility.

## Graphical abstract

**Figure:**
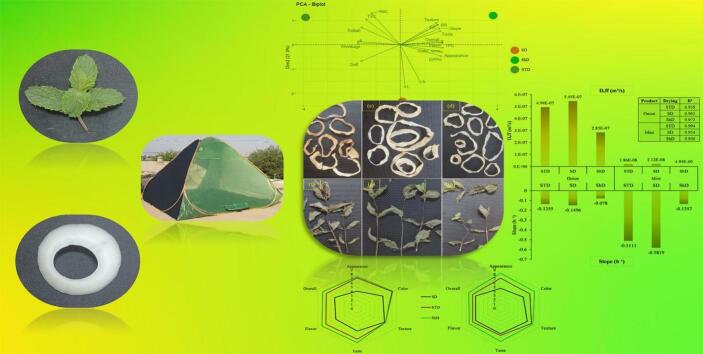

## 1 Introduction

Vegetables and herbs are essential components of human diets, contributing not only to nutrition but also to culinary diversity, flavor, and health-promoting bioactive compounds. Among these, onion (*Allium cepa* L.) and mint (*Mentha piperita* L.) are widely cultivated and consumed for their culinary, medicinal, and aromatic properties (Alinovi et al., 2024). However, their high moisture content renders them highly perishable, leading to rapid microbial spoilage, enzymatic degradation, and extensive postharvest losses. Therefore, efficient drying is critical to extend shelf-life, ensure microbial safety, and maintain the functional and sensory quality of these products, particularly in regions with limited access to cold-chain facilities (Natarajan et al., 2022).

Conventional sun drying (SD) remains the most widely adopted method due to its easiness and low cost. Nevertheless, SD is prone to uneven dehydration, microbial contamination, color and nutrient degradation, and structural collapse which decline its effectiveness (Essalhi et al., 2018). In contrast, shade drying (ShD) is gentler and better preserves color and bioactive compounds. However, it is considerably slower and requires extended drying times, increasing labor demand and operational inefficiency (Bhardwaj et al., 2019a; Reis et al., 2022). Industrial dryers, including hot-air and convective systems, can overcome these limitations but are mostly expensive and inaccessible for smallholder farmers, especially in rural areas. Consequently, drying method selection is a key determinant of both processing cost and product quality. It can also help bridge the gap between traditional and industrial methods (Ge et al., 2026). In recent years, solar-assisted drying technologies have emerged as promising alternatives, combining renewable energy utilization with semi-controlled drying conditions (Boateng, 2024; Shalaby & Bek, 2015). Previous studies have extensively investigated solar and greenhouse drying systems for agro-products, focusing on heat and mass transfer behavior, structural design, and performance optimization (Mhd Safri et al., 2021). Early works demonstrated that greenhouse dryers constructed from PVC frames and UV films can enhance convective heat and mass transfer compared with open sun drying, although limitations such as poor ventilation were reported (Chauhan & Kumar, 2016; Essalhi et al., 2018; Mashonjowa et al., 2013). Subsequent developments introduced modified greenhouse structures, including insulated north-wall systems (Chauhan & Kumar, 2016), reflective surfaces, and integrated solar collectors (Tham et al., 2017a), which improved heat utilization efficiency and drying rates. Modeling approaches have also been applied to predict greenhouse microclimates and drying performance (Fudholi et al., 2016). Morever, advanced configurations incorporating heat pumps or forced airflow have resulted in substantial reductions in relative humidity and drying time, however, they increased structural complexity and operational costs (Tham et al., 2017a). Despite these advances, most existing systems rely on fixed, bulky greenhouse installations designed for stationary use (Chauhan & Kumar, 2016; Essalhi et al., 2018; Fudholi et al., 2016; Mashonjowa et al., 2013; Mhd Safri et al., 2021). In contrast, the present study evaluates a lightweight, portable solar tent dryer with a distinct structural design, with imporved practicality, mobility, and suitability for small-scale or field applications. This portable configuration represents a novel approach that bridges traditional open sun drying and conventional greenhouse technologies.

Overall, this study investigates the drying behavior of onion and mint under SD, STD, and ShD, using thin-layer modeling to describe moisture removal kinetics and estimate effective moisture diffusivity. Quality assessments, including rehydration, color, shrinkage, total phenolic content, antioxidant activity, microbial counts, and sensory evaluation, were conducted to quantify method-dependent impacts. Multivariate analyses, including Pearson correlation and principal component analysis (PCA), were employed to explore the relationships among drying parameters, product quality, and sensory outcomes. By integrating experimental observations with statistical insights, this work provides a comprehensive evaluation of drying strategies and identifies practical, low-cost approaches for preserving the quality of perishable vegetables.

## 2 Materials and methods

### 2.1 Sample preparation

Fresh onion bulbs (*Allium cepa* L.) and mint leaves (*Mentha piperita* L.) were harvested at commercial maturity from local farms in Dezful, Iran. Samples of uniform size, color, and maturity, free from mechanical damage or visible defects, were selected for the study. The vegetables were cleaned to remove soil and other foreign materials, rinsed thoroughly with tap water, and air-dried at ambient temperature to eliminate surface moisture. The onion bulbs were peeled and manually sliced into uniform pieces (3–4 mm) using a sterilized stainless-steel knife to ensure consistency in drying rate and surface area exposure. The initial moisture content of both onion and mint samples was determined by oven drying at 105 ± 1 °C to a constant weight. Measurements were performed in triplicate.

### 2.2 Drying methods and experimental setup

#### 2.2.1 Solar tent drying (STD)

A solar tent dryer (STD) was designed and constructed at the Agricultural Research Station, Dezful district, Iran (32°22′59.1″ N, 48°25′24.9″ E). The system consisted of a lightweight, portable frame covered with a combination of waterproof tarpaulin and a UV-stabilized polyethylene greenhouse film (light transmittance ≈ 90%). This configuration provided both durability and adequate protection of the product from direct ultraviolet radiation while ensuring efficient solar energy utilization.

The drying chamber had overall dimensions of 3.0 × 3.0 × 2.0 m, with a total floor area of 9 m^2^. The inner floor surface was coated with black paint to enhance solar energy absorption and act as a thermal collector. The front side of the tent and the solar collector were inclined at 15° toward the south to maximize solar radiation capture throughout the day. Inside the chamber, four perforated plastic crates (40 × 30 × 20 cm) were arranged vertically with an 18 cm spacing to facilitate uniform airflow and heat distribution. The product samples were loaded onto the crates after prilaminary preparation steps. An exhaust fan (diameter 150 mm, 230 V, 50 Hz, 24 W) was installed at the upper rear side of the drying chamber to ensure continuous air circulation and enhance moisture removal. The fan remained in operation during all drying stages. Prior to loading the samples, the dryer was preheated and stabilized for 60 min to reach steady-state thermal conditions.

**Fig. 1:**
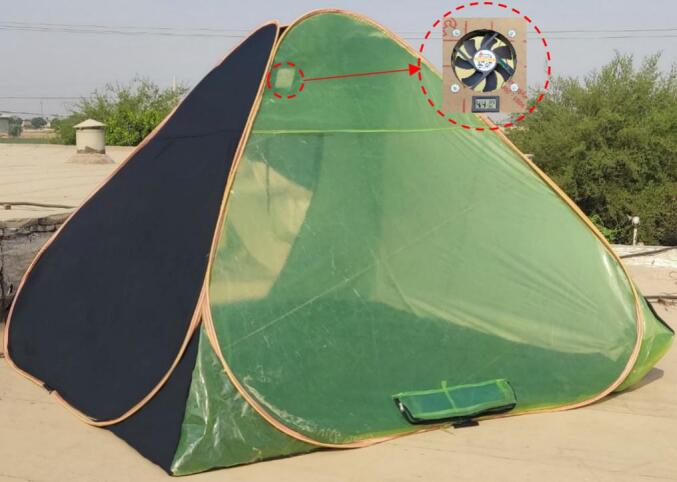
Photographic representation of the solar tent dryer showing the front UV-stabilized polyethylene film for solar capture, and rear exhaust fan with temperature and humidity sensors.

To monitor and record drying conditions, temperature and relative humidity (RH) were measured at the inlet, outlet, and ambient environment. Temperature and relative humidity were measured using a TESTO 174H/174 T data logger (Testo, Lenzkirch, Germany), operating within a temperature range of 0–100 °C (accuracy: ±1 °C; resolution: 0.01 °C) and a relative humidity range of 10–100% (accuracy: ±1%; resolution: 0.1%). The air velocity through the dryer was measured with a Testo 410–1 anemometer (Testo, Lenzkirch, Germany). Samples were distributed in 20 perforated baskets, each containing approximately 200 g of material (10 baskets for onion and 10 for mint), leading to a uniform loading density across all trays. The baskets were evenly placed inside the STD to ensure uniform airflow and heat distribution. In order to eliminate the variability caused by external weather fluctuations, onion and mint samples were dried simultaneously under identical environmental conditions inside the same chamber. Each drying experiment was conducted in triplicate. The samples were loaded at 9:00 a.m. and drying continued until the material reached 10% moisture content (wb). Fig. 1 shows the illustration of the solar tent dryer, and the developed system and experimental configuration for onion and mint samples. This low-cost design provides a simple yet efficient system for rural and small-scale drying applications, combining portability, ease of fabrication, and effective thermal performance.

#### 2.2.2 Sun drying (SD)

For comparison, sun drying was carried out under open-field conditions adjacent to the solar tent dryer. The pretreated samples were spread in a single thin layer on clean perforated plastic baskets (each containing approximately 200 g of material) and exposed directly to sunlight. The baskets were elevated approximately 0.2 m above the ground to ensure adequate air circulation and minimize contamination. Drying continued until the material reached 10% moisture content (wb). No artificial ventilation or protection was provided. The samples were periodically turned every hour to promote uniform drying. Ambient temperature and RH were recorded simultaneously during the drying process using the same sensors as those employed in the solar tent dryer.

#### 2.2.3 Shade drying (ShD)

Shade drying was performed in a naturally ventilated area protected from direct sunlight but exposed to ambient air movement. Samples were placed on perforated trays identical to those used in the sun drying experiment. The trays were positioned under a roofed shed, with proper airflow and protection from rain or debris. Drying continued until the material reached 10% moisture content (wb). Temperature and RH in the shade-drying area were monitored throughout the experiment. Drying was continued until the samples reached constant weight.

### 2.3 Drying kinetics modeling

The drying behavior of onion and mint samples under different drying methods was evaluated by calculating the moisture ratio (MR) and drying rate (DR) at each recorded time interval. The MR was determined using Eq. (1):

(1) $$\;\mathrm{MR}=\frac{M_{t}-M_{e}}{M_{0}-M_{e}}\;\;$$

In this equation, $M_{t}$, $M_{0}$, and $M_{e}$indicate the moisture content (g water/g dry matter) at time *t*, initial, and equilibrium conditions, respectively. Since the $M_{e}$, is relatively small compared with $M_{t}$and $M_{0}$, it was assumed negligible in subsequent calculations.

The drying rate (DR) was determined according to Eq. (2):

(2) $$\begin{array}{cccc} & \mathrm{DR}=\frac{M_{t+\Delta t}-M_{t}}{\Delta t} & & \end{array}$$

where Δt represnts the drying time interval (h).

To describe the drying characteristics, the experimental MR data were fitted to commonly used thin-layer drying models (Table 1**)**. (See Table 2-.)

**Table 1:** Thin-layer drying models used in this study⁎

Model name	Model equation	Reference
Newton	$\mathrm{MR}=\exp\left(-\mathrm{kt}\right)$	[Henderson, 1974]
Page	$\mathrm{MR}=\exp\left(-kt^{n}\right)$	[Page, 1949]
Henderson and Pabis	$\mathrm{MR}=a\;\exp\left(-\mathrm{kt}\right)$	[Henderson & Pabis, 1961]
Logarithmic	$\mathrm{MR}=a\;\exp\left(-\mathrm{kt}\right)+c$	[Yagcioglu et al., 1999]
Midilli–Kucuk	$\mathrm{MR}=a\;\exp\left(-kt^{n}\right)+\mathrm{bt}$	[Midilli et al., 2002]

⁎ t: time (min), k: drying rate constant (min − 1) and a, b, l and n are empirical constants.

**Table 2-:** Thin-layer drying models and corresponding parameter values for onion and mint dried under SD, STD, and ShD conditions.

Models	Model parameters	Onion			Mint		
		STD	SD	ShD	STD	SD	ShD
Newton	k	0.1763	0.2545	0.0651	0.5069	0.5480	0.0927
	R^2^	0.9852	0.9952	0.9952	0.9983	0.9988	0.9886
	RMSE	0.0368	0.0200	0.0200	0.0130	0.0107	0.0384
	χ^2^	0.0014	0.0004	0.0004	0.0002	0.0001	0.0016
Page	k	0.1271	0.2391	0.0498	0.4851	0.5761	0.0533
	n	1.2224	1.0454	1.0900	1.0491	0.9413	1.2220
	R^2^	0.9864	0.9954	0.9954	0.9987	0.9994	0.9990
	RMSE	0.0352	0.0195	0.0195	0.0114	0.0075	0.0113
	χ^2^	0.0014	0.0004	0.0004	0.0002	0.0001	0.0002
Henderson-Pabis	a	1.0369	1.0142	1.0223	1.0088	0.9939	1.0502
	k	0.1852	0.2586	0.0666	0.5110	0.5449	0.0979
	R^2^	0.9863	0.9954	0.9954	0.9984	0.9989	0.9916
	RMSE	0.0354	0.0196	0.0196	0.0126	0.0105	0.0329
	χ^2^	0.0014	0.0004	0.0004	0.0002	0.0001	0.0013
Logarithmic	a	1.0220	0.9996	1.0376	1.0084	0.9860	1.1308
	k	0.1998	0.2716	0.0620	0.5119	0.5647	0.0723
	c	0.0268	0.0199	−0.0242	0.0006	0.0112	−0.1167
	R^2^	0.9903	0.9978	0.9978	0.9984	0.9992	0.9989
	RMSE	0.0298	0.0135	0.0135	0.0126	0.0089	0.0117
	χ^2^	0.0010	0.0002	0.0002	0.0002	0.0001	0.0002
Midilli-Kucuk	a	0.9977	1.0048	0.9939	1.0022	1.0010	0.9967
	k	0.1134	0.2372	0.0489	0.4812	0.5779	0.0586
	n	1.3098	1.0655	1.0906	1.0913	0.9337	1.1502
	b	0.0012	0.0008	−0.0001	0.0021	−0.0003	−0.0011
	R^2^	0.9931	0.9981	0.9981	0.9991	0.9994	0.9996
	RMSE	0.0251	0.0127	0.0127	0.0097	0.0074	0.0068
	χ^2^	0.0008	0.0002	0.0002	0.0002	0.0001	0.0001

The suitability of each model in fitting the experimental data was further evaluated using the coefficient of determination (R^2^), reduced chi-square (χ^2^), and root mean square error (RMSE) according to Eqs. (3, 4 and 5):

(3) $$\begin{array}{cccc} & R^{2}=1-\frac{\sum_{i=1}^{N}{\left(MR_{\exp,i}-MR_{\mathrm{pre},i}\right)}^{2}}{\sum_{i=1}^{N}{\left(MR_{\exp,i}-{\bar{\mathrm{MR}}}_{\exp}\right)}^{2}} & & \end{array}$$

(4) $$\begin{array}{cccc} & \chi^{2}=\frac{\sum_{i=1}^{N}{\left(MR_{\exp,i}-MR_{\mathrm{pre},i}\right)}^{2}}{N-n} & & \end{array}$$

(5) $$\begin{array}{cccc} & \text{RMSE}=\sqrt{\frac{\sum_{i=1}^{N}{\left(MR_{\mathrm{pre},i}-MR_{\exp,i}\right)}^{2}}{N}} & & \end{array}$$

where $MR_{\exp,i}$and $MR_{\mathrm{pre},i}$are the experimental and predicted moisture ratios, Nis the number of data points, and n is the number of model constants. The model providing the highest R^2^ and the lowest χ^2^ and RMSE values was selected as the best-fitting model for describing the drying behavior of each sample under different drying methods.

### 2.4 Effective moisture diffusivity (Dₑff)

The effective moisture diffusivity (Dₑff) represents the combined effect of all moisture transport mechanisms occurring within a drying material. It was determined according to Fick's second law of diffusion, assuming negligible shrinkage, constant diffusion coefficient, and uniform initial moisture distribution throughout the sample.

For an infinite slab geometry, the analytical solution of Fick's equation is given by:

(6) $$\begin{array}{cccc} & \mathrm{MR}=\frac{8}{\pi^{2}}\sum_{n=0}^{\infty }\frac{1}{{\left(2n+1\right)}^{2}}\exp\left[-\frac{{\left(2n+1\right)}^{2}\pi^{2}D_{\mathrm{eff}}t}{4L^{2}}\right] & & \end{array}$$

Here, MR is the moisture ratio (dimensionless), $D_{\mathrm{eff}}$ is the effective moisture diffusivity (m^2^/s), L is the half-thickness of the sample (m), and t is drying time (s). Eq. (6) can be simplified as:

(7) $$\begin{array}{cccc} & \ln\left(\mathrm{MR}\right)=\ln\left(\frac{8}{\pi^{2}}\right)-\frac{\pi^{2}D_{\mathrm{eff}}t}{4L^{2}} & & \end{array}$$

Thus, the effective moisture diffusivity was obtained from the slope of the linear plot of $\ln\left(\mathrm{MR}\right)$ versus drying time (*t*):

(8) $$\begin{array}{cccc} & D_{\mathrm{eff}}=-\frac{4L^{2}}{\pi^{2}}\times \text{slope} & & \end{array}$$

Experimental MR data were fitted to Eq. (7) using Microsoft Excel (Microsoft Corp., USA) and R software (version 4.3.1, R Foundation for Statistical Computing, Vienna, Austria). The resulting $D_{\mathrm{eff}}$ values were expressed in m^2^ s^−1^ and compared among drying methods to evaluate the influence of drying temperature, humidity, and airflow on internal moisture transport.

### 2.5 Physicochemical attributes of dried vegetables

#### 2.5.1 Rehydration ratio / rehydration capacity

Approximately 5 g of dried sample was immersed in 200 mL of distilled water at 40 ± 1 °C for 60 min. The samples were then removed, surface moisture was blotted with tissue paper, and the rehydrated samples were weighed (Bhardwaj et al., 2019a). The rehydration ratio (RR) and rehydration capacity (RC) were calculated using the following equations:

$$\mathrm{RR}=\frac{W_{r}}{W_{d}}$$

$$\mathrm{RC}=\frac{W_{r}-W_{d}}{W_{d}}\times 100$$

where $W_{r}$ and $W_{d}$ are the weights (g) of the rehydrated and dried samples, respectively.

#### 2.5.2 Color analysis

Color measurements were performed using a CM-5 spectrophotometer (Konica Minolta Sensing, Singapore) operating in reflectance mode. Prior to analysis, the instrument was automatically calibrated using its internal white reference standard according to the manufacturer's instructions. Samples were placed directly over the measurement port to ensure full coverage of the aperture and prevent external light interference. The L*, a*, and b* color coordinates, representing lightness, redness/greenness, and yellowness/blueness, respectively, were recorded for each sample. Measurements were conducted at ambient laboratory conditions, and values were obtained directly from the instrument display. The total color difference (ΔE) was calculated according to the following equation

$$\Delta E=\sqrt{{\left(L^{*}-L_{0}^{*}\right)}^{2}+{\left(a^{*}-a_{0}^{*}\right)}^{2}+{\left(b^{*}-b_{0}^{*}\right)}^{2}}$$

where $L_{0}^{*}$, $a_{0}^{*}$, and $b_{0}^{*}$are the color values of the fresh (undried) samples. All measurements were conducted in triplicate.

#### 2.5.3 Shrinkage

Shrinkage was determined by measuring the volume reduction of samples before and after drying. The initial and final volumes were calculated by the liquid displacement method using toluene as the immersion medium to prevent sample absorption. Shrinkage (S, %) was computed as:

$$S\left(\%\right)=\frac{V_{0}-V_{t}}{V_{0}}\times 100$$

where $V_{0}$ and $V_{t}$ are the initial and final sample volumes (cm^3^), respectively. All measurements were performed in triplicate.

#### 2.5.4 Total phenolic content (TPC)

Total phenolic content of dried samples was determined using the Folin–Ciocalteu colorimetric method (Arlee et al., 2025). Approximately 0.5 g of dried sample was extracted with 10 mL of 80% methanol under continuous shaking at 25 °C for 2 h. The extract was centrifuged at 5000 ×*g* for 10 min, and the supernatant was collected.

An aliquot of 0.5 mL extract was mixed with 2.5 mL of 10% Folin–Ciocalteu reagent and incubated for 5 min at room temperature. Then, 2 mL of 7.5% sodium carbonate solution was added, and the mixture was incubated in the dark at 25 °C for 30 min. Absorbance was measured at 765 nm using a UV–Vis spectrophotometer.

TPC was expressed as mg gallic acid equivalent (GAE) per g of dry weight using a calibration curve prepared with gallic acid standards. All analyses were performed in triplicate.

#### 2.5.5 Antioxidant activity (DPPH scavenging)

The free radical scavenging activity of dried samples was evaluated using the DPPH assay (Bachhar et al., 2026). Briefly, 500 μL of sample extract was mixed with 375 μL of ethanol, followed by the addition of 125 μL of 0.15 mM DPPH solution prepared in absolute ethanol. The mixture was incubated in the dark at room temperature for 30 min.

The absorbance was measured at 517 nm using a UV–Vis spectrophotometer. A blank containing distilled water instead of the sample extract was used as the negative control. The percentage of DPPH radical inhibition was calculated as:

$$\%\text{Inhibition}=\frac{A-B}{A}\times 100$$

where A is the absorbance of the control and *B* is the absorbance of the sample solution. All measurements were performed in triplicate.

#### 2.5.6 Microbial assessment

The microbial quality of dried onion and mint samples under different drying methods was evaluated by determining the total viable count (TVC) and yeast and mold counts (YMC) following standard procedures. Approximately 10 g of dried sample was aseptically homogenized in 90 mL of sterile 0.85% saline solution using a stomacher for 2 min. Serial decimal dilutions were prepared. Aliquots were then plated on two microbial media as follows: I) Plate Count Agar (PCA) for total viable count, incubated at 30 ± 1 °C for 48 h, and II) Potato Dextrose Agar (PDA) acidified with 1% tartaric acid for yeast and mold count, incubated at 25 ± 1 °C for 3 days. Colonies were counted, and results were expressed as log CFU/g of dried sample. All analyses were performed in triplicate.

#### 2.5.7 Sensory evaluation

The sensory evaluation involved voluntary participants and complied with the ethical guidelines of the Safiabad Agricultural and Natural Resources Research and Education Center for research involving human participants. Participants were informed about the purpose of the study, the nature of the samples, and their right to withdraw at any time without consequence. Written informed consent was obtained from all participants prior to participation, and all responses were recorded anonymously to ensure confidentiality.

Sensory evaluation of the dried onion and mint samples was conducted to assess the influence of drying methods on overall product acceptability. A semi-trained panel of 10 members (5 males and 5 females, aged 25–45 years) from the food science department participated in the evaluation. Prior to the test, panelists were familiarized with the sensory attributes and scoring method. Samples were rehydrated in distilled water at 25 °C for 10 min, drained, and served at room temperature in coded, odor-free containers. The evaluators were asked to analyze each sample in terms of appearance, color, texture, taste, aroma and overall acceptability (1 = the most dislike to 9 = the most liked). Drinking water was offered for rinsing and palate cleaning between samples. Drinking water was offered for rinsing and palate cleaning between samples. The committee of Technological unit of nutrition and health of Safiabad Agricultural and Natural Resources Research and Education Center approved the procedures applied for organoleptic assessments.

### 2.6 Statistical analysis

All experimental data were statistically analyzed using SPSS software (SPSS Inc., USA). Analysis of variance (ANOVA) was performed at a 5% significance level (*p* < 0.05) to evaluate the effect of drying methods on the measured parameters. Each experiment was performed in triplicate, and results are presented as mean ± standard deviation (SD). In order to compare the means of obtained results, Duncan's multiple range analysis was performed on means and *P* < 0.05 was reported as significant difference. Moreover, principal component analysis (PCA) was performed to provide a comprehensive evaluation of drying performance across different drying methods (SD, STD, and ShD). The analysis integrated all measured variables, including drying kinetics parameters, physicochemical properties, bioactive compounds, antioxidant activity, microbial counts, color indices, and sensory scores. PCA was conducted using standardized data to eliminate scale effects among variables. The resulting principal components were used to visualize relationships among samples and to determine overall similarities or differences among drying treatments.

## 3 Results and discussions

### 3.1 Drying behavior of onion and mint under different drying methods

**Fig. 2:**
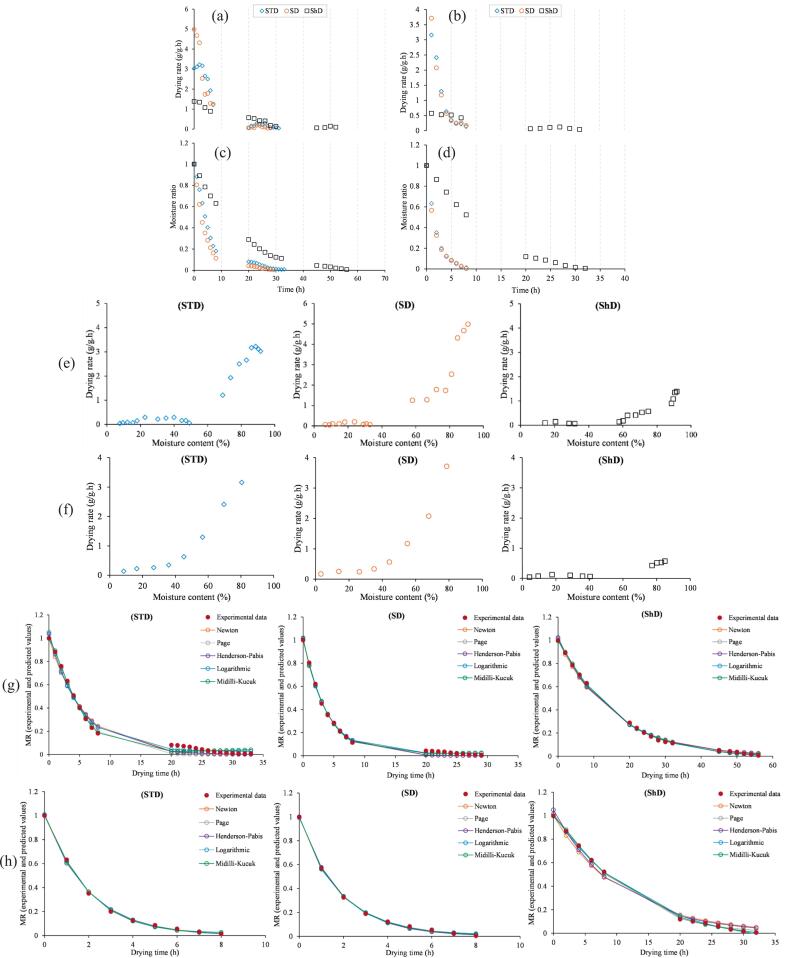
Drying kinetics of onion and mint under SD (sun drying), STD (solar tent drying), and ShD (shade drying). Plots show drying rate versus time for onion (a) and mint (b); moisture ratio versus time for onion (c) and mint (d); drying rate versus moisture content for onion (e) and mint (f); and comparison of experimental and model-predicted moisture ratios over time for onion (g) and mint (h).

The drying behavior of onion and mint showed clear differences across the three drying methods (i.e., solar tent drying (STD), sun drying (SD), and shade drying (ShD), where the extent and pattern of moisture loss were largely governed by drying energy and product characteristics. Fig. 2a–d summarize the changes in moisture ratio (MR) and drying rate (DR) over time.

#### 3.1.1 Moisture ratio and drying rate

Both vegetables experienced a continuous decline in MR throughout drying, but the rate of decline varied considerably between methods. In onion, MR decreased rapidly during the first 6 h under sun drying, dropping from 1.00 to 0.213, which was slightly lower than that observed under STD (0.305 at hour 6). Shade drying exhibited the slowest moisture removal, whrer the MR was still 0.700 at hour 6. After 20 h, MR reached 0.043 in SD, 0.081 in STD, and remained much higher in ShD (0.289), confirming the limited drying potential of shaded conditions.

Mint followed a similar pattern. During the first hour, MR decreased from 1.00 to 0.567 (SD) and 0.632 (STD), showing that sun drying removed moisture slightly faster during the initial stage. By hour 2, both methods produced similar moisture levels (MR ≈ 0.325–0.351), demonstrating comparable drying efficiency under strong sunlight. Shade drying again was slower, with MR still 0.865 at hour 2. At the end of primary drying (hour 8), SD achieved the lowest MR (0.005), followed by STD (0.014) and ShD (0.523), illustrating the markedly slower dehydration under shade. (Bhardwaj et al., 2019b) compared shade drying with an indirect forced-convection solar dryer enhanced with sensible heat storage and phase-change materials for Valeriana jatamansi rhizomes. The solar dryer reduced the drying time to about 5 days, whereas shade drying required roughly 14 days. They observed rapid moisture loss at the beginning due to surface evaporation, followed by a gradual decline in drying rate as moisture migrated from the interior to the surface. Similar drying behavior has also been reported by (Rabha & Muthukumar, 2017) and (Shalaby & Bek, 2015).

Drying rate curves showed a distinct falling-rate period for all products and drying methods, indicating that internal moisture diffusion controlled the process once surface water was removed. For onion, the highest initial DR was observed under sun drying, reaching 4.99 g/g.h at hour 0–1, slightly above STD (3.03 g/g.h) and far above the ShD (1.39 g/g.h). Throughout the first 6 h, SD consistently showed equal or greater DR values than STD, confirming the stronger drying potential of direct solar radiation during peak sunlight. Mint displayed the same early drying behavior. At hour 1, SD and STD exhibited DR values of 3.72 and 3.16 g/g.h, respectively, both much higher than ShD (0.58 g/g.h). After hour 2, their DR profiles became similar (≈ 2.0–2.4 g/g·h). This suggests that for lightweight leafy materials, sun drying and tent drying perform similarly under strong sunlight, whereas shade drying sharply restricts water removal. Across both vegetables, ShD produced consistently lower DR values, often below 1.0 g/g.h. Our drying rate results are consistent with the findings of (Yahya et al., 2016), who documented DR values ranging from 0.15 to 2.3 kg/h (average 1.33 kg/h) when using a solar dryer, and from 0.55 to 2.8 kg/h (average 1.93 kg/h) in a solar-assisted heat pump drying system. Similarly, (Tham et al., 2017b) studied drying of Java tea (*Orthosiphon aristatus*) and Sabah snake grass (*Clinacanthus nutans*) in a solar greenhouse dryer equipped with a heat pump. They found that sun drying gave a higher initial drying rate, most likely because free surface moisture evaporated quickly under direct sunlight. However, the total drying time was similar for both methods. They also observed that samples dried with the heat-pump system maintained a constant drying rate over a wider moisture range (MR ≈ 0.9–0.2), whereas sun-dried samples entered the falling-rate stage shortly after reaching the peak drying rate. This suggests that assisted drying systems can maintain higher drying efficiency for a longer period than open sun drying.

As indicated in Fig. 2c-d, plotting DR against moisture content revealed a clear decline in drying rate as moisture decreased, supporting the absence of a constant-rate period. For onion, DR at high moisture content (>0.7) was highest in SD, intermediate in STD, and lowest in ShD; for example, at MC ≈ 0.90, DR was approximately 4.68 g/g.h (SD), 3.11 (STD), and 1.39 (ShD). As moisture decreased below 0.25, DR values overlapped and became very small (<0.3 g/g.h), indicating that internal diffusion and structural resistance limited water movement regardless of drying energy. Mint showed a similar pattern: at high MC (∼0.8), DR was around 3.7 g/g.h in SD and 3.16 g/g.h in STD and less than 0.6 g/g.h in ShD. At low MC (<10%), DR dropped to near-zero values (e.g., 0.13–0.170 g/g.h in STD/SD and < 0.1 in ShD). In a similar attempt, (Arbaoui et al., 2025) modified a solar greenhouse into a solar dryer and evaluated its performance during the summer for drying *Myrtus communis* L. They reported that drying inside the solar dryer accelerated moisture removal, with the herbs reaching their minimum moisture content after about 8 h. In contrast, samples dried outside showed much slower moisture reduction and required approximately 22 h to reach their lowest moisture level. A similar pattern of moisture reduction was reported by (Rathore & Panwar, 2010) during grape drying in a hemicylindrical walk-in solar tunnel dryer, and by (Pita-Garcia et al., 2025) during cocoa drying in a solar–electric hybrid system combining sun drying (SD) with auxiliary heating.

In general, sun drying produced the highest drying rates for both products during the early stages, especially during strong sunlight. Tent drying was slightly slower at the beginning but became comparable afterward, benefiting from its semi-closed structure that retains heat and reduces losses. Shade drying was consistently the slowest, with prolonged high MR values and low DR throughout. On the other hand, among the studied vegetables, mint dried much faster than onion under both STD and SD. For instance, MR fell from 1.00 to 0.20 in mint within 3 h (SD), whereas onion reached only 0.45 (SD) and 0.63 (STD) in the same period. This can be attributed to mint's thin, leafy structure and high surface-area-to-volume ratio, which enhanced mass transfer. Onion slices, with multilayered tissues and higher initial moisture, imposed greater internal resistance to water diffusion. Under shaded conditions, however, both products dried very slowly, confirming the dominant role of drying energy over product characteristics under low-heat conditions.

#### 3.1.2 Mathematical modeling

Mathematical modeling was applied to describe and interpret the drying kinetics of onion and mint under three drying methods (STD, SD, and ShD). Five thin-layer models (i.e., Newton, Page, Henderson–Pabis, Logarithmic, and Midilli–Kucuk) were fitted to the experimental moisture ratio data, and their performance was evaluated using the coefficient of determination (R^2^), root mean square error (RMSE), and chi-square (χ^2^). The fits were also visually assessed through plots comparing experimental and predicted MR values (Fig. 2e-f). Overall, all five models properly fitted the experimental MR values, however, the parameter values and goodness-of-fit indicators varied between products and drying methods, reflecting the notable differences imposed by product structure and drying intensity.

Across all models, sun drying generally produced higher drying-rate constants (k) than solar tent drying and especially shade drying, consistent with the experimentally observed faster moisture removal under direct sunlight. For onion, the Newton model yielded k values of 0.2545 (SD) > 0.1763 (STD) > 0.0651 (ShD), while mint showed a similar trend with 0.5480 (SD) > 0.5069 (STD) > 0.0927 (ShD). Particularly, the gap between SD and STD for mint was small, reflecting their nearly equivalent drying rates observed experimentally. Shade drying consistently resulted in smaller k values for both products (e.g., 0.0651 for onion and 0.0927 for mint), indicating much slower diffusion under low-energy conditions.

For both products, introducing additional empirical parameters generally improved model flexibility and fit compared with the simple Newton equation. The Page model showed the most substantial enhancement, with mint under SD achieving the highest accuracy (R^2^ = 0.9994) and onion under both SD and ShD showing identical, strong fits (R^2^ = 0.9954). The behavior of the *n* exponent consistently reflected increased curvature where drying has lower intensity, as can be seen in higher *n* values for ShD in both mint (1.2220) and onion (1.0900). The Henderson–Pabis model offered performance similar to Newton, but its *a* parameter captured minor deviations from experimental values, especially under ShD where higher a values (1.0502 for mint) corresponded to the longer, slower speed of moisture removal seen under low-temperature drying. The Logarithmic model further improved fit quality, particularly for onion under SD and ShD (R^2^ = 0.9978) and mint under SD (R^2^ = 0.9992), with its *c* parameter accounting for early-stage curvature or lag effects. Negative *c* values under ShD for both products indicated the expected initial delay in moisture diffusion associated with low thermal and convective driving forces.

Across all models, the Midilli–Kucuk model provided the best performance for both products and all drying methods, with the highest R^2^ values and the lowest RMSE and χ^2^. For onion, SD and ShD achieved identical R^2^ values (0.9981) with RMSE of 0.0127, while STD also performed strongly (R^2^ = 0.9931). Mint showed even stronger fits, with R^2^ = 0.9994 (SD) and 0.9996 (ShD), and RMSE values of 0.0068 under shade drying. The b parameter, representing the linear component of the model, was positive under faster drying conditions (e.g., 0.0021 for mint–STD), but tended toward zero or became slightly negative under slower methods, again reflecting the extended falling-rate period under ShD. Comparable trends were noted by (Patel & Panwar, 2022) during their assessment of Cucumis callosus dried in a natural-convection solar tunnel system. In their study, most drying models achieved R^2^ values above 0.95, indicating strong agreement between predicted and experimental moisture data. Among the evaluated models, the Midilli equation provided the best overall fit for kachri, with an R^2^ of 0.971, RMSE of 0.0632, and χ^2^ of 0.0046. The authors concluded that both the Midilli and Page models offered superior predictive performance, characterized by high modeling efficiency and low error statistics, and were therefore the most suitable for describing the drying behavior of the product. In a similar study, (Chauhan & Kumar, 2018) examined thermal modeling and drying kinetics of gooseberries dried in a north-wall-insulated greenhouse dryer. They reported high coefficients of determination (R^2^ = 0.98, 0.99, 0.99, 0.99, and 0.95) for the Lewis, Page, Henderson and Pabis, Logarithmic, and Midilli–Kucuk models, respectively, which are comparable to the R^2^ values obtained in the present study. (Demiray et al., 2017) also examined the drying kinetics of onion (*Allium cepa* L.) slices under convective and microwave drying. They fitted several mathematical models to the experimental moisture data and evaluated model performance using standard statistical criteria. Their results showed that all models achieved R^2^ values above 0.90, confirming an acceptable and reliable goodness of fit across drying conditions. Moreover, comparable changes in the parameters of thin-layer drying models have also been reported for other products, including cocoa (Pita-Garcia et al., 2025), chamomile (Amer et al., 2018) as well as longan and banana dried either under direct sunlight or inside a solar greenhouse dryer (Janjai et al., 2009).

Overall, the differences in model parameters between onion and mint highlight the role of tissue structure: mint, with its delicate leafy morphology, responds more sharply to drying intensity, as reflected in more pronounced variations in k and n values. In contrast, onion exhibits smoother and more uniform declines in MR, leading to slightly tighter clusters of goodness-of-fit indices across drying methods. These modeling outcomes align closely with the experimental observations and provide a strong basis for predicting drying performance under similar conditions.

### 3.2 Effective moisture diffusivity (Dₑff)

**Fig. 3:**
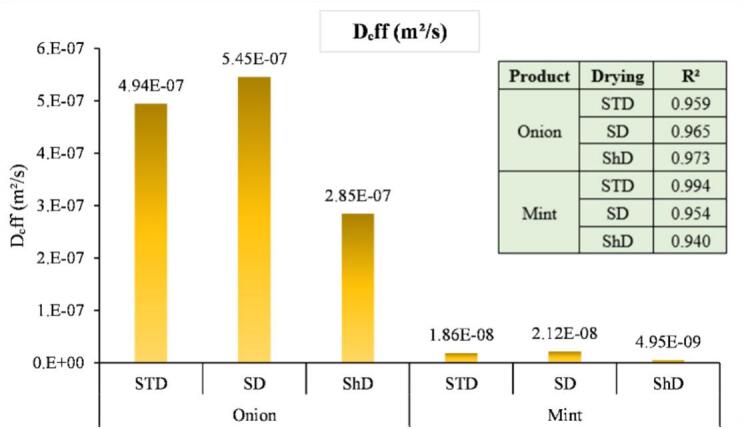
Effective moisture diffusivity (Dₑff) of onion and mint subjected to SD, STD, and ShD.

The effective moisture diffusivity (Dₑff) of onion and mint, calculated from the slope of the linearized drying curves, varied between products and drying methods (Fig. 3). In onion, Dₑff was highest under sun drying (5.45 × 10^−7^ m^2^/s), slightly lower in solar tent drying (4.94 × 10^−7^ m^2^/s), and markedly lower in shade drying (2.85 × 10^−7^ m^2^/s), indicating that direct solar radiation and the semi-enclosed tent structure promoted faster internal moisture transport, whereas limited heat under shade drying restricted water diffusion. The difference between sun drying and solar tent drying was minimal, showing that both methods achieved nearly similar internal diffusivity. Mint exhibited considerably lower Dₑff values than onion (2.12 × 10^−8^ m^2^/s in sun drying, 1.86 × 10^−8^ m^2^/s in solar tent drying, and 4.95 × 10^−9^ m^2^/s in shade drying), but dried much faster overall. This apparent discrepancy arises because mint's thin, leafy structure and high surface-area-to-volume ratio allow rapid water removal from the surface, making external mass transfer the dominant factor. In contrast, thicker onion slices require longer internal diffusion paths, even though Dₑff is higher. These findings align with the observations of (Mehta et al., 2018), who analyzed the falling-rate drying stage and applied Fick's diffusion model to estimate moisture diffusivity in fish dried using a solar dryer. They reported an effective diffusivity of 1.53 × 10^−7^ m^2^/s, noting that this value falls well within the typical range for food materials (10^−12^ to 10^−8^ m^2^/s). (Pita-Garcia et al., 2025) reported an effective moisture diffusivity of 4.12 × 10^−7^ m^2^/s for cocoa dried using a hybrid system (sun drying combined with solar–electric drying). They also noted that increasing the drying temperature led to higher Dₑff values, indicating faster internal moisture movement at elevated temperatures.

Across both vegetables, Dₑff correlated well with the slope of the ln(MR) vs. time plots, confirming that internal moisture diffusion governed the falling-rate period. Shade drying imposed significant resistance to internal water movement, whereas sun and solar tent drying achieved nearly similar diffusivity, providing sufficient energy for rapid dehydration. The R^2^ values for all linear fits were above 0.94, indicating excellent agreement between the experimental data and the diffusion model.

### 3.3 Physicochemical attributes of dried vegetables

#### 3.3.1 Rehydration ratio/rehydration capacity

**Fig. 4:**
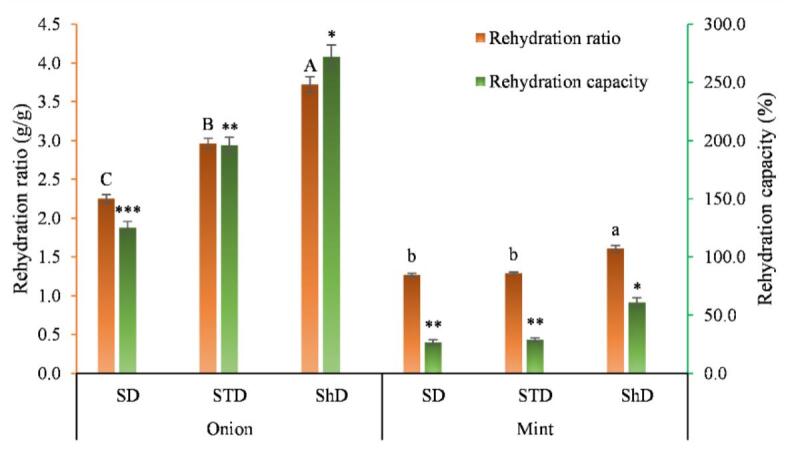
Rehydration ratio (RR), and rehydration capacity (RC) of onion and mint subjected to SD, STD, and ShD.

The rehydration characteristics of the samples under different drying methods are presented in Fig. 4. As shown, both RR and RC of onion slices were significantly affected by the drying technique (*p* < 0.05). Shade-dried samples exhibited the highest rehydration ratio (3.722 g/g) and rehydration capacity (272.2%), followed by solar-tent drying (RR: 2.962 g/g; RC:196.2%) and sun drying (RR: 2.252 g/g; RC: 125.2%). The superior rehydration ability of shade-dried samples can be attributed to minimized thermal stress during dehydration, which helps preserve textural integrity and avoid excessive shrinkage and structure hardening. Gentle drying promotes the formation of a more porous matrix that facilitates water diffusion during rehydration. In contrast, sun drying exposes the tissue to elevated surface temperatures and uneven heat distribution, leading to structural collapse and reduced capacity to absorb water. These observations align with reports that rehydration behavior is a strong indicator of structural preservation in thermally sensitive vegetables.

Mint samples possessed markedly lower RC and RR compared with onion due to the delicate strucuture of leafy tissues, which are more prone to structural damages during dehydration. Shade drying resulted in the highest RR (1.6087 g/g) and RC (60.87%), that was significantly higher than solar-tent drying (RR: 1.29 g/g; RC:26.67%) and sun drying (RR: 1.27 g/g; RC:26.67%) (*p* < 0.05). The gentle drying conditions in ShD induces less damage to the integrity of plant texture, thereby reducing shrinkage and maintaining the capillary structure required for water absorption. On the other hand, SD and STD expose the mint leaves to elevated temperatures, leading to rapid moisture loss, and collapse of the lamina, which in turn decline their rehydration potential. The comparable RC values of SD and STD indicate that leafy herbs are more prone to thermal damages than vegetables with thicker tissues. Similarly, (Seremet Ceclu, Botez, Nistor, Andronoiu and Mocanu, 2016) examined the effects of hot-air and microwave drying at various temperatures and found that higher drying temperatures markedly reduced the rehydration ratio. For example, hot-air drying at 70 °C resulted in a rehydration ratio of 79.81 ± 0.3%, compared with 88.97 ± 0.3% at 60 °C. (Bhardwaj et al., 2019c) reported that the notable differences in the rehydration ratio of medicinal Himalayan plants dried using traditional methods, compared with those dried in the novel solar drying systems investigated in their study, could be attributed to large variations in drying temperature during the processes. They also noted that rhizomes dried at a uniform temperature exhibited a porous structure and a loose outer layer, whereas rhizomes dried at higher temperatures developed a collapsed outer layer.

#### 3.3.2 Color analysis

**Fig. 5:**
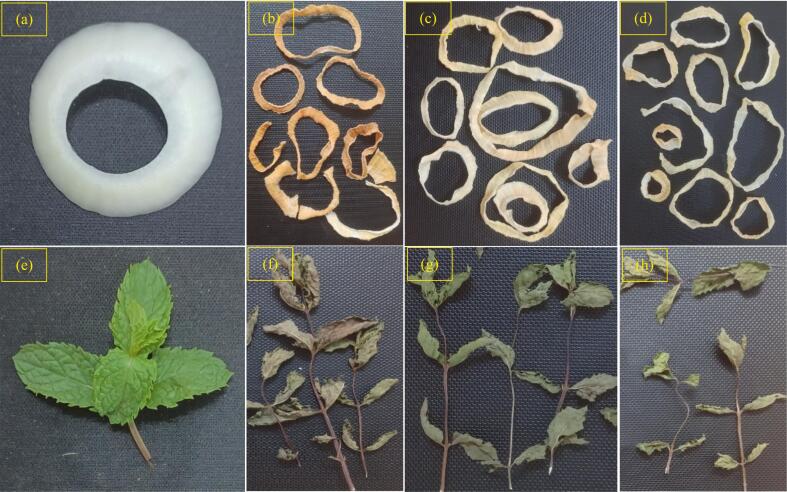
Visual appearance of fresh and dried onion and mint samples under different drying methods: a) fresh onion, b) SD-onion, c) ShD-onion, d) STD-onion; e) fresh mint, f) SD-mint, g) ShD-mint, h) STD-mint.

Color was noticeably affected by the drying method in both onion and mint samples (Table 3), and the instrumental measurements aligned well with the visual changes seen in the images (Fig. 5). Fresh onion showed a bright white appearance (L* = 92.00 ± 2.20, a* = −3.50 ± 0.53, b* = −0.88 ± 1.13). However, when dried, the extent of color deviation varied strongly among the drying methods. Sun-dried onion experienced the most intense discoloration, with a clear loss of lightness (83.78 ± 2.68) and a strong shift toward redness (a* = 5.56 ± 0.88) and yellowness (b* = 26.89 ± 2.80). This changes were well coordinated with the visible yellow-brown tone (Fig. 5b) and was reflected in the highest ΔE value (30.34). Shade drying, in contrast, preserved the onion's natural color, maintaining relatively high lightness (88.63 ± 2.67) and only mild shifts in the a* and b* values. Its ΔE (6.44) was the lowest among all dried samples, consistent with the cleaner, less browned appearance (Fig. 5d). The solar tent dryer produced intermediate results (L* = 87.25 ± 3.11 and ΔE = 12.15), showing lighter browning than the sun-dried samples but still more color change than shade drying.

**Table 3-:** Color parameters of onion and mint sampls dried under SD, STD, and ShD methods.

Sample	Drying method	L*	a*	b*	∆E
Onion	Fresh onion	92.00 ± 2.20 a	−3.50 ± 0.53 d	−0.88 ± 1.13 d	
	SD	83.78 ± 2.68 c	5.56 ± 0.88 a	26.89 ± 2.80 a	30.34
	STD	87.25 ± 3.11 b	0.00 ± 1.07 b	9.75 ± 1.16 b	12.15
	ShD	88.63 ± 2.67 b	−1.25 ± 0.46 c	4.13 ± 1.36 c	6.44
Mint	Fresh mint	62.38 ± 3.81 b	−18.50 ± 1.41 d	27.88 ± 4.64 a	
	SD	63.89 ± 4.94 ab	0.11 ± 2.52 a	11.44 ± 1.74 c	24.87
	STD	67.63 ± 1.92 a	−3.63 ± 2.00 b	16.75 ± 2.55 b	19.30
	ShD	64.25 ± 3.41 ab	−6.25 ± 1.39 c	13.88 ± 2.64 bc	18.70

Mint behaved in a similar way, although the type of color change differed due to its naturally dark-green pigment profile. Fresh mint displayed a strong negative a* value (−18.50 ± 1.41) and a high b* value (27.88 ± 4.64). Sun drying caused the most severe degradation in the color, where the a* sharply shifted toward the red axis (0.11 ± 2.52) and b* declined to less than half of the fresh value (11.44 ± 1.74ᶜ). Visually, these samples appeared brownish-green and flaccid and this was confirmed by a large ΔE of 24.87. Both the solar tent dryer and shade drying retained the green color more effectively. Their a* values (−3.63 ± 2.00 and − 6.25 ± 1.39, respectively) still showed a loss of greenness but remained substantially closer to the fresh sample. Correspondingly, their ΔE values (19.30 and 18.70) were lower than those of the sun-dried samples. In the images, the shade-dried mint retained a slightly darker, more natural green than the tent-dried sample, reflecting the small differences in the instrumental data. These results indicate the importance of low-radiation drying environments for maintaining the natural color of aromatic and vegetable tissues. Rerpotadely, drying generally cause a reduction in the lightness of the dried plants (Arslan & Musa Özcan, 2010; Janjai et al., 2009; Pita-Garcia et al., 2025). For instance, (Pita-Garcia et al., 2025) found a remarkable darker color in the cocoa beans dried in diffrend solar drying systems where the lightness was decreased from 53.18 to values between 31.09 and 35.39. However, the highest decline was found in the samples dried under direct sunlight. Upon comparing the onion slices dried in convective oven, microwave oven and under sun, (Arslan & Musa Özcan, 2010) reported tha drying across all methods significantly reduce the lightness (L*) of the onion slices compared with the fresh sample. The greatest redcution in L* occurred in samples dried in the hot-air oven, indicating that higher temperatures and prolonged exposure intensify color degradation. Their findings underscore that harsher thermal conditions accelerate browning and overall discoloration during onion drying. (Janjai et al., 2009) also evaluated the performance of a PV-ventilated solar greenhouse dryer for drying peeled longan and banana. They reported a pronounced decrease in lightness (L*) in naturally sun-dried samples, whereas solar-dried counterparts showed smaller color changes compared with fresh samples. (Bhardwaj et al., 2019c) also designed a novel solar dryer and reported that, compared with traditional drying, solar dryers are more effective in preserving the color attributes of medicinal plants in the Western Himalayan region.

#### 3.3.3 Shrinkage

**Fig. 6:**
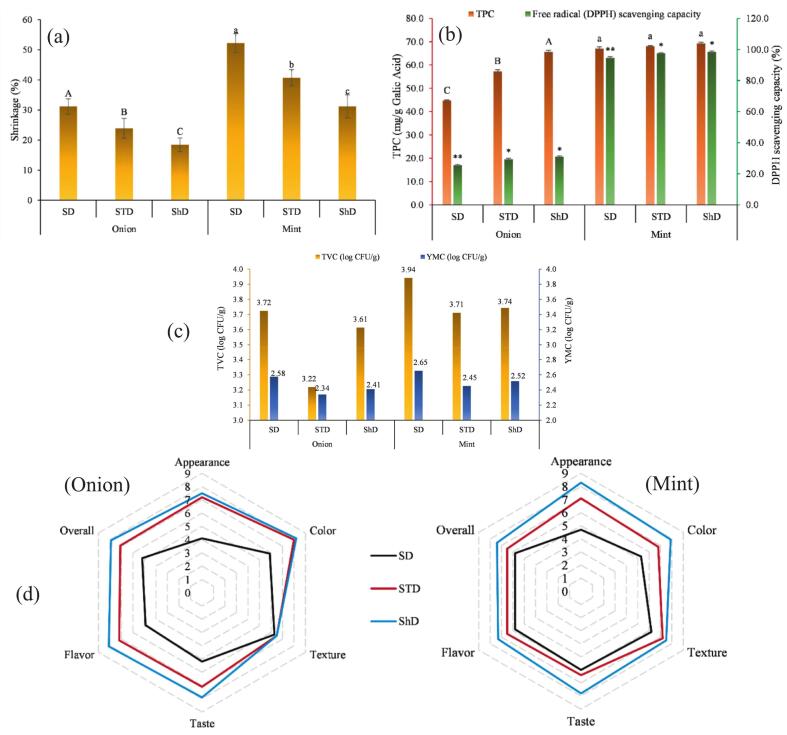
quality attributes of onion and mint dried under SD, STD, and ShD: (a) shrinkage, (b) total phenolic content (TPC) and DPPH scavenging activity, (c) total viable count (TVC) and yeast and mold count (YMC), and (d) sensory scores

Shrinkage measurements further illustrated the structural changes induced by the different drying techniques (Fig. 6**a**). In onion slices, shrinkage ranged from 18.45% in shade drying to 23.92% in solar-tent drying and 31.17% under sun drying. The relatively low shrinkage observed in shade-dried samples reflects the milder thermal exposure and slower moisture removal, conditions that help preserve the cellular framework and limit collapse. In contrast, direct sun drying resulted in the greatest volume loss, suggesting more pronounced deformation of the tissue during dehydration. A similar pattern was evident in mint, though with generally higher shrinkage values due to the thin, delicate leaf structure. Sun-dried mint showed the most severe shrinkage (52.26%), followed by solar-tent drying (40.72%) and shade drying (31.18%). These trends are in clear agreement with the rehydration characteristics reported earlier: treatments that caused lower shrinkage also exhibited superior rehydration performance. For instance, shade-dried onion, showing the least shrinkage, displayed the highest rehydration ratio (3.722 g/g) and rehydration capacity (272.2%), whereas sun-dried onion, with the greatest volume reduction, showed the lowest values (RR 2.252 g/g, RC 125.2%). Mint samples followed the same general pattern, with shade drying yielding both lower shrinkage and markedly higher rehydration capacity (60.87%) compared with sun drying (26.67%). Taken together, these results confirm that the extent of structural preservation during drying strongly influences later water uptake, and they further highlight the advantage of shade 0and solar-tent drying over conventional sun drying. The greater impact of sun drying on the shrinkage of dried plant materials was also reported by (Pita-Garcia et al., 2025) during cocoa bean drying either under direct sun or in a hybrid solar–electric drying system, where sun-dried beans showed a significant reduction in both length and equivalent radius.

#### 3.3.4 Total phenolic content (TPC) and antioxidant activity (DPPH scavenging)

Drying method had a remarkable influence on the total phenolic content (TPC) and free radical scavenging capacity (DPPH%) of both onion and mint (Fig. 6**b**). In onion samples, TPC ranged from 44.71 to 65.67 mg GAE/g, with the lowest value recorded under sun drying (44.71 mg/g) and the highest under shade drying (65.67 mg/g). The solar tent dryer resulted in an intermediate value (57.33 mg/g). A similar trend was observed for DPPH scavenging activity, which increased from 25.55% in sun drying to 29.37% in the solar tent dryer and 31.10% in shade drying. These results indicate that enhanced phenolic retention is closely associated with stronger antioxidant potential in onion, reflecting the sensitivity of phenolic compounds to thermal and photooxidative degradation under direct sunlight. (Pita-Garcia et al., 2025) also examined the phenolic content and antioxidant capacity of cocoa beans dried in different solar drying systems (i.e., direct sun drying, solar-electric hybrid drying, and a combination of both systems). They similarly reported that a significant reduction in TPC was observed with processes that included SD. These results suggest that exposure of cocoa beans to sunlight is a critical factor affecting phenolic compound content. On the other hand, antioxidant capacity decreased significantly under all drying processes, with the greatest reduction again observed in treatments that included SD. Similarly, (Amer et al., 2018) investigated how drying methods affect the volatile oil content (%) of fresh and dried chamomile processed by solar drying and open sun drying. They reported that chamomile dried in the solar dryer retained a higher essential oil percentage than samples dried under direct sunlight. This difference was attributed to the more controlled and uniform temperature conditions in solar drying, whereas uneven heating during natural sun drying may promote greater loss of volatile compounds. (Seifu et al., 2018), examined the impact of different drying temperatures on the quality attributes of onion. They reported that increasing oven-drying temperatures significantly reduced key bioactive components in onion samples, including vitamin C and pyruvic acid.

Mint showed higher absolute levels of both TPC and DPPH activity compared with onion, consistent with its naturally rich phenolic composition. The TPC of mint increased from 65.10 mg/g in sun drying to 68.17 mg/g in the solar tent dryer and 70.36 mg/g in shade drying. Antioxidant activity followed the same pattern where, DPPH scavenging ranged from 94.63% in sun drying to 97.55% in the solar tent dryer and 98.49% in shade drying. The higher antioxidant activity of mints dried under STD and ShD methods further highlights the protective effect of reduced direct radiation and moderated temperatures. The aligned trends between TPC and DPPH% in both products demonstrate a strong positive relationship between phenolic preservation and antioxidant capacity. Shade drying consistently provided the highest retention, followed by the solar tent dryer, while conventional sun drying caused the greatest degradation. (Arbaoui et al., 2025) also investigated the physicochemical changes of *Myrtus communis* L. dried using different solar greenhouse dryers and open sun drying. They reported that direct sun drying caused the greatest losses in total flavonoids and sugar content. In addition, the IC₅₀ values of samples dried in the greenhouse dryer were lower than those of sun-dried samples, indicating stronger antioxidant activity in the greenhouse-dried herbs.

#### 3.3.5 Microbial assessment

The microbial quality of dried onion and mint samples varied noticeably among the drying methods, reflecting the influence of temperature, airflow, and drying duration on microbial inactivation (Fig. 6**c**). In onion, the total viable count (TVC) ranged from 1.66 × 10^3^ to 5.30 × 10^3^ CFU/g, corresponding to 3.219 to 3.725 log CFU/g, while yeast and mold counts (YMC) were between 2.19 × 10^2^ and 3.76 × 10^2^ CFU/g (2.340 to 2.575 log CFU/g). Solar drying consistently yielded the highest microbial loads, particularly for TVC (5.30 × 10^3^ CFU/g) and YMC (3.76 × 10^2^ CFU/g), a pattern commonly attributed to prolonged exposure to fluctuating temperatures and the potential for environmental contamination during outdoor drying. In contrast, shade drying produced intermediate values (TVC 4.11 × 10^3^ CFU/g; YMC 2.58 × 10^2^ CFU/g), whereas the solar tent dryer reduced microbial counts more effectively (TVC 1.66 × 10^3^ CFU/g; YMC 2.19 × 10^2^ CFU/g), likely due to its higher internal temperature, lower drying time, and reduced exposure to open air and insects.

A similar trend was observed in mint samples, although absolute counts were slightly higher than onion, likely reflecting the larger surface area, higher initial microbial load, and more intricate leaf microstructure of herbs. TVC values ranged from 5.15 × 10^3^ to 8.75 × 10^3^ CFU/g (3.712 to 3.942 log CFU/g), and YMC from 2.83 × 10^2^ to 4.50 × 10^2^ CFU/g (2.452 to 2.653 log CFU/g). Again, solar drying produced the highest microbial load (TVC 8.75 × 10^3^ CFU/g; YMC 4.50 × 10^2^ CFU/g), whereas the tent dryer and shade drying produced substantially lower values. Across both products, tent drying consistently limited microbial counts more effectively than traditional sun drying, reinforcing the advantage of semi-controlled drying systems that combine solar energy with improved enclosure and airflow. The yeast and mold levels in all samples remained within the safety thresholds established by the International Commission on Microbiological Specifications for Foods (ICMS), staying below the recommended limits of 3.0 log CFU/g for yeasts and 4.0 log CFU/g for molds. Besides, in all dried samples it remained below the typical range reported for sun- and shade-dried vegetables and herbs (generally 10^3^–10^5^ CFU/g), demonstrating that the drying operations were effective in lowering microbial growth (Akinwotu et al., 2025; García et al., 2010; Savitha et al., 2025). The consistently higher values in the solar-dried samples highlight the importance of protective drying structures in preventing recontamination and reducing the survival of heat-resistant microorganisms.

#### 3.3.6 Sensory evaluation

Organoleptic results revealed clear distinctions among the sensorial attributs of dried onion and mint samples (Fig. 6**d**). For onion, panelists scored sun-dried samples remarkably lower in most attributes, particularly in appearance (4.1), color (5.9), and overall acceptability (5.2). These results align with the color degradation, higher shrinkage (31.17%), and reduced rehydration ability (RR: 2.252 g/g; RC: 125.2%) previously obtained for sun-dried onion, all of which can contribut to the less appealing visual and textural quality after rehydration. In contrast, solar-tent drying and shade drying yielded markedly superior organoleptic attributes. Shade-dried onion received the highest score for appearance (7.5), color (8.2), flavor (8.1), and overall acceptability (7.9), followed by solar-tent drying (overall score: 7.1). These results reflect the better color retention (lowest ΔE: 6.44), reduced shrinkage (18.45%), and enhanced rehydration characteristics (RR: 3.722 g/g; RC: 272.2%) of shade-dried onion. A similar pattern was evident for mint where, the Sun-dried mint samples received the lowest scores, particularly for appearance (4.7) and color (5.3), consistent with their higher shrinkage (52.26%), greater color change (ΔE: 24.87), and more noticeable surface darkening. Conversely, shade-dried mint achieved the most favorable evaluations, with appearance (8.3), color (7.9), flavor (7.3), and overall acceptability (7.4) rated highest among the treatments. This enhancement in sensorial features are well coordinated the superior retention of phenolics (TPC: 70.36 mg GAE/g), the highest antioxidant activity (98.49% DPPH inhibition), and the lowest shrinkage (31.18%), all indicating the better protection of structural and bioactive quality. Solar-tent dried mint also performed well in different evaluated attributes (overall: 6.5), reflecting the moderated thermal treatment of the semi-enclosed system. The oragnoleptic results confirmed that minimizing the thermal and oxidative stress, particularly in shade drying, and solar-tent drying, properly preserved the visual, structural, and flavor quality of onion and mint, and produced products more appealing to consumers. Similar changes in the organoleptic attributes of banana and longan upon drying in different solar-based drying systems were reported by (Janjai et al., 2009). Their sensory evaluation results showed that longan and banana dried inside a PV-ventilated solar greenhouse dryer had higher liking scores in terms of appearance, color, texture, flavor, taste, and overall acceptance compared with sun-dried products. A significant difference between the liking scores of solar-dried and sun-dried longan and banana was observed at the 0.05 level.

### 3.4 Multivariate relationships among instrumental, chemical and sensory outcomes

**Fig. 7:**
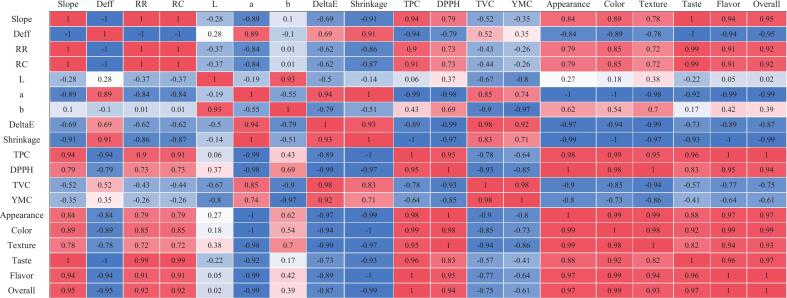
Pearson correlation matrix of various attributes of mint samples dried using SD, STD, and ShD methods.

**Fig. 8:**
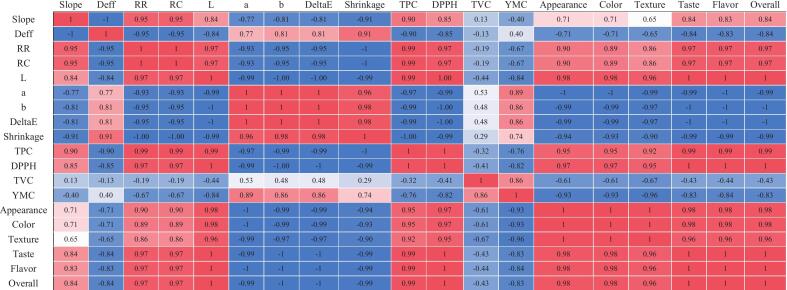
Pearson correlation matrix of various attributes of onion samples dried using SD, STD, and ShD methods.

To explore the relationship among instrumental, chemical and sensory outcomes across different drying methods, Pearson correlation matrices were calculated separately for mint and onion (Fig. 7, Fig. 8). However, since the analyzed variables describe different physical and chemical properties, the correlations were interpreted carefully, and only relationships with clear physical meaning were discussed. As can be observed, for both mint and onion, several consistent patterns were observed. A first group of variables representing overall quality retention of the dried products. They showed strong positive correlations. Rehydration indices (RR and RC) correlated closely with total phenolic content and antioxidant activity (mint: RR–TPC ≈ 0.90, RR–DPPH ≈ 0.73; onion: RR–TPC ≈ 0.99). Sensory scores followed the same trend. For example, overall acceptability correlated strongly with TPC (mint ≈ 1.00; onion ≈ 0.99) and RR (mint ≈ 0.92; onion ≈ 0.97). These results indicate that drying treatments preserving structure also preserved chemical quality and sensory properties. A second group corresponded to structural degradation indicators. Shrinkage and total color difference (ΔE) were highly positively correlated with each other (mint ≈ 0.93; onion ≈ 0.98) and strongly negatively correlated with quality variables such as TPC (mint ≈ −1.00; onion ≈ −1.00) and sensory scores (generally < −0.94). The redness parameter (a*) behaved similarly, showing strong positive correlations with shrinkage (mint ≈ 1.00; onion ≈ 0.96). These relationships confirm that more severe structural damage is associated with pigment degradation and lower perceived quality.

Drying intensity indicators also followed clear trends. The drying slope and effective diffusivity (Dₑff) were negatively related to quality parameters and positively related to structural damage markers. For example, in mint samples slope correlated negatively with shrinkage (≈ −0.91) and positively with TPC (≈ 0.94), while Dₑff showed the opposite pattern (≈ +0.91 with shrinkage and ≈ −0.94 with TPC). Similar trends were observed for onion. This indicates that faster moisture transport tends to intensify structural changes and quality loss. Microbial counts (TVC and YMC) were strongly correlated with each other (mint ≈ 0.98; onion ≈ 0.86) but showed weaker or inconsistent correlations with most physicochemical and sensory variables. This suggests that microbial levels were influenced mainly by drying hygiene and environmental exposure rather than intrinsic product quality.

### 3.5 Principal component analysis (PCA)

PCA was performed to integrate all physicochemical, structural, microbiological, and sensory variables and to visualize the multivariate differences among the drying methods. For both mint and onion, two principal components (PC) effectively represented the dataset, explaining **100% of the total variance**. PC1 captured around **78.7%** of variations in mint and **89.2%** in onion, indicating that most quality attributes responded consistently to the drying method. PC2 explained the remaining **21.2%** and **10.8%,** respectively.

**Fig. 9:**
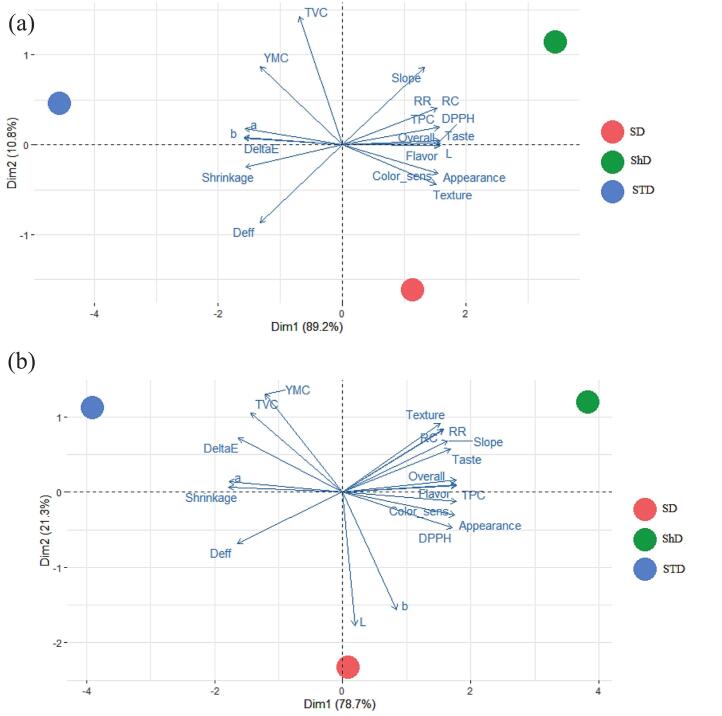
PCA biplots showing multivariate differences among drying methods: a) onion, b) mint.

The PCA plot for mint revealed a clear separation among the drying methods along PC1 (Fig. 9). Sun-dried samples were located on the negative side of PC1, primarily due to the higher shrinkage, greater color degradation (a*, b*, ΔE), and lower sensory scores. Shade-dried samples clustered on the positive side of PC1, strongly affected by higher TPC, DPPH activity, and better appearance, flavor, and overall acceptability. Solar tent–dried mint positioned in an intermediate location but remained significantly closer to shade drying than to sun drying. Its position reflected a favorable balance between color retention, antioxidant activity, and sensory quality. A similar pattern was observed for onion. PC1 seperated the sun drying, characterized by higher shrinkage, increased color change, and lower sensory attributes, from the more quality-preserving shade drying. Shade-dried samples aligned positively with variables such as L*, TPC, DPPH activity, and all sensory attributes. Solar tent–dried onion again located between the other two methods, but toward the favorable quadrant shared with shade drying. Its position reflects higher rehydration capacity, better color stability, moderate microbial loads, and improved sensory properties compared with sun drying. Across both products, PCA consistently showed that **solar tent drying forms a distinct and favorable cluster**, close to shade drying.

## 4 Conclusion

This study systematically compared solar tent drying (STD), sun drying (SD), and shade drying (ShD) for onion (*Allium cepa* L.) and mint (*Mentha piperita* L.), through evaluating drying kinetics, quality attributes, microbial safety, and sensory properties. SD showed the highest initial drying rates, with effective moisture diffusivity reaching 5.45 × 10^−7^ m^2^/s in onion, while ShD was the slowest due to limited thermal input. Shade drying preserved the highest total phenolic content (up to 70.36 mg GAE/g in mint) and antioxidant activity (98.49% DPPH), as well as superior rehydration capacity. However, its prolonged drying time limits practical application. In contrast, STD achieved a balanced performance, maintaining good color retention (ΔE 12.15 for onion), moderate shrinkage, substantial bioactive preservation, and lower microbial loads (onion TVC 1.66 × 10^3^ CFU/g) compared with SD, while requiring considerably shorter drying durations than ShD. Sensory scores further supported the overall quality advantages of STD and ShD over SD. Overall, although shade drying maximized certain quality attributes, solar tent drying emerged as a practical, low-cost, and energy-efficient alternative that effectively balances drying efficiency with preservation of nutritional, structural, and sensory quality. These findings demonstrate the potential of simple solar-assisted drying systems for sustainable small-scale vegetable processing and postharvest loss reduction.

## CRediT authorship contribution statement

**Seid Reza Falsafi:** Writing – review & editing, Writing – original draft, Supervision, Software, Project administration, Investigation, Conceptualization. **Ramin Gooruee:** Investigation. **Hamid Reza Gazor:** Visualization, Validation.

## Funding

This research received no specific grant from any funding agency in the public, commercial, or not-for-profit sectors.

## Declaration of competing interest

The authors declare that they have no known competing financial interests or personal relationships that could have appeared to influence the work reported in this paper.

## Data Availability

Data will be made available on request.
